# The impact of partner autonomy constraints on women's health-seeking across the maternal and newborn continuum of care

**DOI:** 10.1016/j.eclinm.2022.101715

**Published:** 2022-10-31

**Authors:** Shannon N. Wood, Robel Yirgu, Celia Karp, Meseret Zelalem Tadesse, Solomon Shiferaw, Linnea A. Zimmerman

**Affiliations:** a Department of Population Family and Reproductive Health, Johns Hopkins Bloomberg School of Public Health, Baltimore, MD, USA; b Addis Ababa University School of Public Health, Addis Ababa, Ethiopia; c Maternal, Child, and Nutrition Directorate, Ethiopia Federal Ministry of Health, Addis Ababa, Ethiopia

**Keywords:** Maternal and child health, Intimate partner violence, Reproductive coercion, Partner involvement, Care-seeking behaviors, AAU, Addis Ababa University, ANC, antenatal care, FMoH, Federal Ministry of Health, IPV, intimate partner violence, JHBSPH, Johns Hopkins Bloomberg School of Public Health, MNH, maternal and newborn health, PMA, Performance Monitoring for Action, PNC, postnatal care, RC, reproductive coercion

## Abstract

**Background:**

Gendered health inequities impede women's reproductive autonomy over the life course. Pregnancy is a critical time point for assessing inequities and partners are integral actors in the achievement or impediment of women's and children's health during this time.

**Methods:**

Among a nationally representative cohort of Ethiopian women 5–9 weeks postpartum with data collected from October 2019–September 2020, this study aimed to 1) understand the prevalence and interplay of partner-related autonomy constraints (intimate partner violence (IPV), reproductive coercion (RC), lack of encouragement from seeking antenatal care (ANC), and lack of encouragement from seeking postnatal care (PNC), and 2) examine the impact of autonomy constraints on the maternal and newborn health (MNH) continuum of care.

**Findings:**

Sixty percent of women experienced a partner-related autonomy constraint prior to or during pregnancy. Approximately 20% of women were not encouraged to seek antenatal care and postpartum care, respectively, whereas fewer women experienced IPV during pregnancy (12.3%) and RC (11.0%). Less than one in five women completed the MNH continuum of care. Lack of encouragement of ANC and PNC were associated with decreased care-seeking at every point across the MNH continuum of care. Lack of encouragement of ANC (aOR = 0.45; p = 0.05) and of PNC (aOR = 0.16; p < 0.001) were associated with reductions in completing the continuum.

**Interpretation:**

Partner engagement, interventions, and messaging are critical to enhance MNH care-seeking behaviors.

**Funding:**

This work was supported, in whole, by the 10.13039/100000865Bill & Melinda Gates Foundation [INV 009466]. Under the grant conditions of the Foundation, a Creative Commons Attribution 4.0 Generic License has already been assigned to the Author Accepted Manuscript version that might arise from this submission.

**Research in context**

**Evidence before this study**

Prior evidence points to the positive and negative roles that partners can play in women's health and well-being. On July 05, 2022, we searched PubMed using the terms ("intimate partner violence" OR "reproductive coercion" OR "autonomy constraint" OR "discouragement") AND ("maternal health" OR "maternal and child health" OR "child health") AND ("sub-Saharan Africa"). This search yielded 24 articles, 12 of which did not specify a maternal or child health outcome and seven did not describe care-seeking behaviors. Four studies focused on intimate partner violence as a primary exposure and found associated significant decreases in timely antenatal care and facility delivery. One qualitative study, conducted in Mozambique, described the role of partners in influencing maternal health care-seeking.

**Added value of this study**

Using a nationally representative cohort of pregnant women in Ethiopia, we found that sixty percent of women experienced a partner-related autonomy constraint, inclusive of intimate partner violence, reproductive coercion, discouragement from seeking antenatal care, or discouragement from seeking postnatal care, prior to or during pregnancy. Less than one in five women completed the maternal and newborn health continuum of care. While autonomy constraints generally occurred in isolation, all constraints were associated with reductions in care-seeking behaviors across the continuum of care: reproductive coercion with infant immunization; IPV during pregnancy with facility-based delivery; and partner discouragement of antenatal care or postnatal care with all outcomes.

**Implications of all available evidence**

Less than one in five women completed the maternal and newborn health continuum of care in Ethiopia and male partners are critical actors in maternal and child health-seeking behaviors. The current research and practice base must extend beyond intimate partner violence to better understand the roles that male partners play in encouraging or discouraging care-seeking at this critical stage of the life course.

## Introduction

Gendered social and health inequities impede women's sexual and reproductive autonomy over the life course.^1^ Such inequities can act across multiple socioecological levels, however, the partner/couple level is particularly critical to women's reproductive autonomy given the dyadic nature of reproductive and pregnancy decision-making. While supportive partners may bolster women's health and happiness, non-supportive, controlling, and/or violent partners can hamper women's health and well-being.^2^^,^^3^ Partner-related constraints on women's reproductive autonomy have most often been framed to focus on the most severe constraints and outcomes, including intimate partner violence (IPV), where globally one in three women will face IPV over the course of their lifetime,^4^ or reproductive coercion (RC), where male partners intentionally act against their female partner's reproductive intentions.^5^ Such autonomy constraints are known to incur profound sexual, reproductive, and mental health consequences,^6^^,^^7^ including increased risk of mortality.^8^

Pregnancy is a critical time for assessing partner-related autonomy constraints given potential impacts to both mother and baby. Global estimates indicate that 2–14% of women experience IPV during pregnancy,^9^^,^^10^ and such violence is associated with increased odds of miscarriage, premature labor, low birthweight, and maternal depression.^11^, ^12^, ^13^, ^14^ While the associations between IPV and maternal and newborn health (MNH) outcomes have been extensively studied, less is known about the health impact of other related partner autonomy constraints, such as RC. Further, male partners may inhibit women's care-seeking through expressed opinions and verbal pressures without explicit perpetration of violence—these more indirect autonomy constraints, including discouragement or lack of engagement in pregnancy-related care-seeking, can still hamper women's health through limiting access to necessary medical services.^15^

In Ethiopia, as with other similar resource-constrained settings, MNH care-seeking behaviors remain suboptimal. Limited care-seeking from antenatal care (ANC) through delivery and postnatal care (PNC) has been linked to increased maternal and newborn morbidity and mortality.^16^ Despite an extensive public healthcare system and the widespread service availability within communities through the Health Development Army, or Health Extension Workers (HEWs), utilization of services remains low.^17^ National data from Ethiopia in 2019 indicate that less than half of women complete the four or more ANC visits recommended by the Ethiopian Federal Ministry of Health (FMoH), with numbers substantially differing between urban (66.9%) and rural (35.4%) areas.^18^ Further, approximately half of pregnant women deliver at home and half do not receive any postnatal care.^18^ Similarly, less than half of infants receive the recommended vaccinations for BCG and polio within the required six-weeks after birth.^18^ To date, research surrounding barriers to care-seeking have largely focused on health systems and access constraints, while recognizing that women's personal situations, including their relationships, may influence access to and willingness to attend healthcare visits. Moreover, while individual interventions improve maternal mortality, it is widely recognized that strategies targeting multiple socioecological levels must be utilized concurrently to achieve international development goals.^16^

Against this backdrop, this study aimed to 1) understand the prevalence and interplay of partner-related autonomy constraints during and immediately prior to pregnancy; and 2) examine the impact of partner-related autonomy constraints on care-seeking across the MNH continuum of care among a nationally representative cohort of pregnant Ethiopian women.

## Methods

### Study design

This analysis is situated within the Performance Monitoring for Action (PMA)-Ethiopia cohort study, a collaboration between Johns Hopkins Bloomberg School of Public Health (JHBSPH), Addis Ababa University (AAU), and the FMoH. PMA-Ethiopia collects data on a cohort of 2873 pregnant women at pregnancy, six-weeks, six-months, and one-year postpartum. Enrollment and baseline survey occurred from October–December 2019. Households within six regions of Ethiopia, covering roughly 90% of the population, were first identified from a census. All women ages 15–49 were screened for potential pregnancy (n = 2879 eligible from screening; n = 2855 complete baseline survey; completion rate = 99.2%). The multistage sample selection of enumeration areas selected using probability proportional to size ensures a nationally representative sample.

This analysis utilizes linked baseline and six-week postpartum data. Six-week interviews were conducted from November 2019–September 2020, with a pause for COVID-19 restrictions from April–July 2020 (n = 1644 interviews pre-COVID; n = 913 interviews during COVID).

All surveys were translated prior to interview and interviews conducted in local language of participant preference; for culturally diverse regions, local translators assisted with interviews. Oral consent was obtained from all participants prior to interview. The full protocol for PMA Ethiopia is detailed elsewhere.^19^ Institutional Review Board approval was obtained at both JHSPH and AAU; protocols follow best practices for violence research^20^ and surveys conducted during COVID followed JHSPH safe human subjects research protocols.

### Participants

Analyses were restricted to the 2388 women who were pregnant at baseline and completed subsequent six-week data collection (retention = 82.7%); retention was non-differential by partner-related autonomy constraints. Analyses were further restricted to women who were currently married or living with a partner as if married given focus on partner-related autonomy constraints with complete data (n = 1886).

### Measures

Primary exposures were four partner-related autonomy constraints: 1) RC, 2) IPV, 3) lack of partner encouragement for ANC, and 4) lack of partner encouragement for PNC. Partner-related autonomy constraint items were pilot tested using cognitive interviewing prior to survey implementation—no issues with comprehension and interpretation of items were indicated.

RC was measured via a four-item pregnancy coercion sub-scale modified from the RC Scale that was developed, validated, and refined in the United States.^21^, ^22^, ^23^ Psychometric analyses indicated one latent construct (eigenvalue = 1.84; factor loadings>0.4) and moderate reliability (Cronbach's alpha = 0.69). Past-year RC items comprised “said he would leave you if you didn't get pregnant”; “told you he would have a baby with someone else if you didn't get pregnant”; “took away your family planning or kept you from going to the clinic”; and “hurt you physically because you did not agree to get pregnant”. A fifth item was measured within PMA Ethiopia (told you not to use family planning), however, previous analyses indicated that item wording lacked clear motivation and thus functioned differently from other RC items; therefore, it was not utilized for the present analysis. All items were assessed at baseline interview when women were pregnant (i.e., examined experience prior to pregnancy). RC was analyzed as a binary variable, with affirmative response to any of the four RC items indicative of any past-year RC experience.

IPV during pregnancy was measured via the 10-item Revised Conflict and Tactics Scale,^24^ which asks about specific violence behaviors per best practices for violence research.^20^ IPV during pregnancy was measured at the six-week postpartum interview to ensure the most thorough measurement of violence over the course of the entire pregnancy. IPV was analyzed as binary, with affirmative response to any of the ten items indicative of IPV experience during pregnancy.

Lack of partner encouragement of ANC was measured via a single item assessed at the six-week postpartum interview: “Did your partner encourage you to go to the clinic for antenatal care?” Response options included: “Yes”; “No, did not encourage”; and “No, actively discouraged”, where “No, did not encourage” and “No, actively discouraged” were combined into a single binary response to indicate lack of encouragement due to small cell sizes. Notably, <1% of participants indicated “actively discouraged”, with the majority of affirmative responses indicating “did not encourage.”

Similarly, lack of partner encouragement of PNC was measured via a single item assessed at the six-week postpartum interview: “Did your partner encourage you to go to the clinic for postnatal care?” Response options and distributions mirrored those of lack of partner encouragement of ANC. Similar to lack of encouragement of ANC, this dichotomized variable largely reflects a lack of encouragement from partners, with overt discouragement being much less common (<1% of women).

Additionally, given substantial overlap between lack of partner encouragement of ANC and lack of partner encouragement of PNC, a categorical variable was created to understand potential dose–response associations of lack of encouragement with care-seeking throughout the MNH continuum of care. The categorical variable was defined as encouragement of ANC and PNC (coded as 0); partner encouragement of ANC or PNC (coded as 1); and lack of partner encouragement for both ANC and PNC (coded as 2).

Primary outcomes examine a range of care-seeking behaviors across the MNH continuum of care, specifically, 1) complete ANC contact (four or more visits), 2) facility-based delivery, 3) receipt of any PNC by six-weeks postpartum, and 4) infant immunization (receipt of both Bacillus Calmette–Guérin (BCG) and polio vaccination); all outcomes were assessed dichotomously and utilized standard assessments.^17^^,^^19^ Additionally, a binary variable was created to examine completion of the MNH continuum of care (completion of all four care-seeking behaviors vs. three or fewer care-seeking behaviors). All outcome data collection occurred at the six-week postpartum interview.

A number of sociodemographic covariates that could confound the associations between partner-related agency constraints and care-seeking behaviors were considered based on theory and prior literature, including, residence (urban/rural); age (15–19, 20–29, 30–39, 40–49); marital status (married/living with a partner); education (never attended, primary, secondary or higher); parity (nulliparous, 1, 2–3, 4+), religion (Orthodox, Muslim, Protestant/Other); and polygynous marriage (yes/no). Additionally, given split of the six-week interview into pre-COVID and during-COVID periods, a pre/post variable was created to assess potential confounding by six-week interview period.

### Statistical analysis

Descriptive statistics examined the distribution of sociodemographic characteristics. Venn diagrams classified overlap in partner-related autonomy constraints; cross-tabulations, bivariate, and multivariable logistic regressions further assessed correlations between partner-related autonomy constraints. Bivariate and multivariable logistic regressions were then run separately between each autonomy constraint and MNH continuum of care outcomes. Latent class and clustered analyses were explored to examine potential clustering and interplay in exposures, however, initial analyses indicated that exposures often occurred in isolation; as such, subsequent regression analyses analyzed exposures individually, with the exception of lack of partner encouragement of ANC and PNC, for which a categorical variable was created to examine a dose–response relationship. Sociodemographic covariates were selected for inclusion within multivariable models based on p < 0.1 from bivariate models. Potential confounders were assessed for multi-collinearity; as such, only urban/rural residence, parity, and education were retained within adjusted models. All analyses were conducted in STATA 16, with statistical significance set at p < 0.05; the svy command accounted for stratification during sample selection, clustering within enumeration areas, and the application of survey weights (including loss-to-follow-up weighting).

### Role of the funding source

The funding source played no role in the study design; collection, analysis, and interpretation of data; in writing the report; or in the decision to submit the paper for publication.

Quantitative data are available upon request from www.pmadata.org.

All authors read and approved the final version of this manuscript and agreed to submit for publication.

## Results

Sociodemographic characteristics, partner-related autonomy constraints, and MNH continuum of care outcomes are presented in Table 1. Recently pregnant Ethiopian women predominantly resided within rural regions (78.3%), were married (97.4%), and had one or more children (77.0%). The majority were between ages 20–29 (54.0%) and had primary or lower education (81.2%).

**Table 1:** Sociodemographic characteristics, partner-related autonomy constraints, and MNH continuum of care outcomes of recently pregnant Ethiopian women (n = 1886).

Sociodemographic characteristics	Unweighted n (%)	Weighted n (%)
Residence		
Urban	695 (36.9)	409 (21.7)
Rural	1191 (63.2)	1477 (78.3)
Age		
15–19	161 (8.5)	192 (10.2)
20–29	1063 (56.4)	1019 (54.0)
30–39	591 (31.3)	593 (31.5)
40–49	71 (3.8)	82 (4.3)
Marital status		
Married	1820 (96.5)	1836 (97.4)
Living with a partner	66 (3.5)	50 (2.6)
Education		
Never attended	732 (38.8)	787 (41.7)
Primary	682 (36.2)	745 (39.5)
Secondary or higher	472 (25.0)	354 (18.8)
Parity		
Nulliparous	464 (24.6)	434 (23.0)
1	398 (21.1)	377 (20.0)
2–3	514 (27.3)	505 (26.8)
4+	510 (27.0)	569 (30.2)
Religion		
Orthodox	853 (45.2)	711 (37.7)
Muslim	562 (29.8)	643 (34.1)
Protestant/other	471 (25.0)	532 (28.2)
Husband/partner has other wives	164 (8.7)	171 (9.1)
**Exposures:partner-related autonomy constraints**		
RC	204 (10.8)	206 (11.0)
IPV during pregnancy	213 (11.3)	232 (12.3)
Lack of partner encouragement of ANC	377 (20.0)	426 (22.6)
Lack of partner encouragement of PNC	381 (20.2)	407 (21.6)
Combined lack of partner encouragement		
Encouraged for both ANC and PNC	1345 (71.3)	1286 (68.2)
Lack of encouragement of ANC or PNC	324 (17.2)	366 (19.4)
Lack of encouragement of ANC and PNC	217 (11.5)	233 (12.4)
**Outcomes: MNHcontinuum ofcare**		
Complete ANC	910 (48.3)	814 (43.2)
Facility delivery	1165 (61.8)	1043 (55.3)
Any PNC by six-weeks postpartum	1045 (55.4)	940 (49.9)
Infant immunization^a^	873 (47.9)	728 (39.9)
Complete continuum of care^a^	443 (24.3)	318 (17.4)

MNH = maternal and newborn health; RC = reproductive coercion; IPV = intimate partner violence; ANC = antenatal care; PNC = postnatal care.

a Among women with infants still living at six-week interview (n = 1824).

Prevalence of each partner-related autonomy constraint ranged between affecting one in ten women to just over one in five: past-year RC (11.0%), IPV during pregnancy (12.3%), lack of partner encouragement of PNC (21.6%) and lack of partner encouragement of ANC (22.6%). MNH care-seeking behaviors across the continuum of care were more common—just over half of women delivered within a facility (55.3%) and just under half of women reported any PNC by six-weeks postpartum (49.9%). Fewer women completed ANC (four or more visits; 43.2%) or reported infant immunization by six-weeks postpartum (39.9%). Less than one in five women (17.4%) completed all four care-seeking behaviors.

**Fig. 1:**
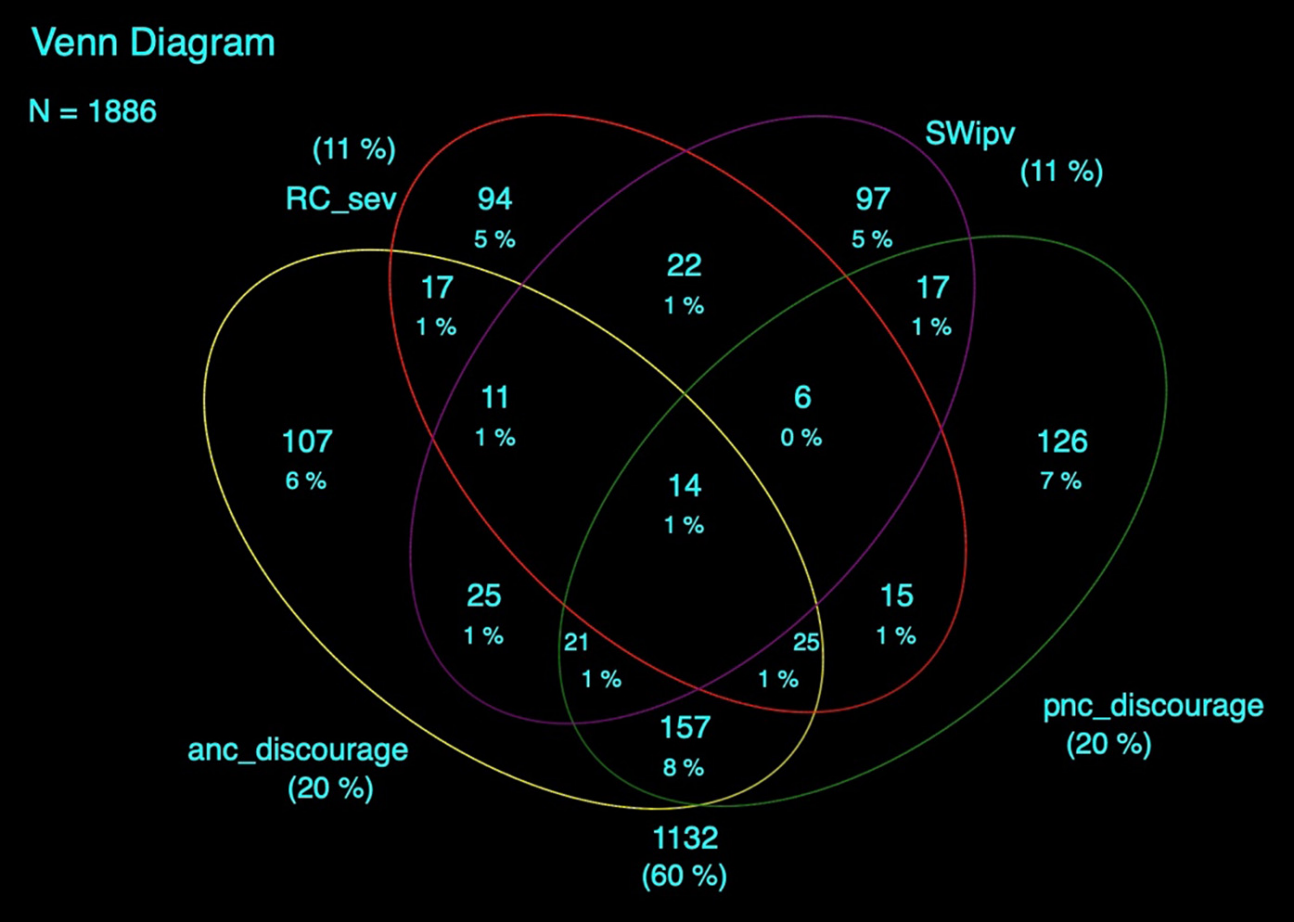
**Venn diagram of partner-related autonomy constraints, unweighted.** RC_sev = RC; SWipv = IPV during pregnancy; anc_discourage = lack of encouragement of ANC; pnc_discourage = lack of encouragement for PNC.

Sixty percent of recently pregnant women experienced at least one partner-related autonomy constraint prior to or during pregnancy (Fig. 1). These experiences often occurred in isolation; however, the most common overlap was lack of encouragement of both ANC and PNC (11%).

Table 2 presents the interplay of partner-related autonomy constraints via results of multivariable logistic regression models. Experience of RC prior to pregnancy was associated with three-fold increased odds of IPV experience during pregnancy (aOR = 3.35; 95% CI = 2.14–5.23; p < 0.001). RC was also associated with two-fold increased odds of lack of partner encouragement of ANC (aOR = 2.01; 95% CI = 1.24–3.28; p = 0.01), however, was not significantly associated with lack of partner encouragement of PNC. IPV during pregnancy was associated with increased odds of lack of partner encouragement of ANC (aOR = 1.82; 95% CI = 1.12–2.96; p = 0.05); bivariate models showed comparable associations between IPV and lack of partner encouragement of PNC, however, this relationship was attenuated when adjusted for sociodemographic characteristics. Lack of partner encouragement of ANC and lack of partner encouragement of PNC showed the strongest correlations of all partner-related autonomy constraints—women who experienced lack of encouragement of ANC had over six-fold increased odds of experiencing lack of encouragement of PNC (aOR = 6.76; 95% CI = 4.81–9.50; p < 0.001), and vice-versa.

**Table 2:** Distribution and interplay of partner-related autonomy constraints (n = 1886), weighted.

	RC	IPV during pregnancy	Lack of partner encouragement of ANC	Lack of partner encouragement of PNC
RC				
No (%)		**10**.**4**	**21**.**1**	20.7
Yes (%)		**28**.**3 ∗∗∗**	**35**.**0 ∗∗**	28.8
OR (95% CI)		**3**.**42 (2**.**20, 5**.**33) ∗∗∗**	**2**.**01 (1**.**25, 3**.**25) ∗∗**	1.55 (0.88, 2.74)
aOR (95% CI)^a^		**3**.**35 (2**.**14, 5**.**23) ∗∗∗**	**2**.**01 (1**.**24, 3**.**28) ∗∗**	1.42 (0.81, 2.51)
IPV during pregnancy				
No (%)	**9**.**0**		**20**.**9**	**20**.**4**
Yes (%)	**25**.**2 ∗∗∗**		**34**.**5 ∗∗**	**29**.**6 ∗**
OR (95% CI)	**3**.**41 (2**.**20, 5**.**33) ∗∗∗**		**1**.**98 (1**.**23, 3**.**20) ∗∗**	**1**.**63 (1**.**02, 2**.**60) ∗**
aOR (95% CI)^a^	**3**.**34 (2**.**14, 5**.**23) ∗∗∗**		**1**.**82 (1**.**12, 2**.**96) ∗**	1.52 (0.96, 2.40)
Lack of partner encouragement of ANC				
No (%)	**9**.**2**	**10**.**4**		**11**.**9**
Yes (%)	**16**.**9 ∗∗**	**18**.**8 ∗∗**		**54**.**8 ∗∗∗**
OR (95% CI)	**2**.**01 (1**.**25, 3**.**25) ∗∗**	**1**.**98 (1**.**23, 3**.**20) ∗∗**		**8**.**99 (6**.**56, 12**.**30) ∗∗∗**
aOR (95% CI)^a^	**2**.**05 (1**.**27, 3**.**31) ∗∗**	**1**.**84 (1**.**13, 2**.**99) ∗∗**		**6**.**76 (4**.**81, 9**.**50) ∗∗∗**
Lack of partner encouragement of PNC				
No (%)	9.9	**11**.**1**	**13**.**0**	
Yes (%)	14.6	**16**.**9 ∗**	**57**.**4 ∗∗∗**	
OR (95% CI)	1.55 (0.87, 2.74)	**1**.**63 (1**.**02, 2**.**60) ∗**	**8**.**99 (6**.**56, 12**.**30) ∗∗∗**	
aOR (95% CI)^a^	1.45 (0.83, 2.54)	1.52 (0.96, 2.41)	**6**.**69 (4**.**76, 9**.**41) ∗∗∗**	

Row percentages reported; OR/aOR from svy: logistic regression model.

p-value ∗<0.05; ∗∗<0.01; ∗∗∗<0.001; bold indicates p < 0.05.

RC = reproductive coercion; IPV = intimate partner violence; ANC = antenatal care; PNC = postnatal care.

a Adjusted for urban/rural residence, parity, education.

Associations between partner-related autonomy constraints and completion of MNH continuum of care outcomes are presented in Table 3. Approximately one in four women (29.0%) who experienced past-year RC had their child immunized, compared to 41.2% immunization among women without RC experience (aOR = 0.53; 95% CI = 0.34–0.84; p = 0.01). RC prior to pregnancy was not associated with any other outcomes along the MNH continuum of care. Among all MNH outcomes, IPV during pregnancy was only associated with facility-based delivery; women who experienced IPV reported a 50% reduction in delivering within a facility (aOR = 0.50; 95% CI = 0.32–0.78; p = 0.01).

**Table 3:** Associations between partner-related autonomy constraints and MNH continuum of care outcomes (n = 1886), weighted.

	Complete ANC	Facility delivery	PNC by six-weeks postpartum	Infant immunization^a^	Entire continuum of care^a^
RC					
No (%)	43.7	56.2	50.2	**41.2**	18.1
Yes (%)	39.2	48.0	47.5	**29.0 ∗∗**	12.1
OR (95% CI)	0.83 (0.56, 1.23)	0.72 (0.46, 1.11)	0.90 (0.58, 1.40)	**0**.**58 (0.37, 0.91) ∗**	0.63 (0.37, 1.06)
aOR (95% CI)^b^	0.89 (0.62, 1.27)	0.70 (0.45, 1.10)	0.96 (0.62, 1.48)	**0.53 (0.34, 0.84) ∗∗**	0.66 (0.39, 1.14)
IPV during pregnancy					
No (%)	43.9	**57.5**	50.3	40.9	18.0
Yes (%)	37.9	**39.6 ∗∗**	46.7	33.0	13.3
OR (95% CI)	0.78 (0.48, 1.26)	**0.48 (0.31, 0.76) ∗∗**	0.87 (0.54, 1.38)	0.71 (0.46, 1.12)	0.70 (0.36, 1.35)
aOR (95% CI)^b^	0.82 (0.51, 1.33)	**0.50 (0.32, 0.78) ∗∗**	0.96 (0.60, 1.56)	0.70 (0.47, 1.07)	0.81 (0.43, 1.53)
Lack of partner encouragement of ANC					
No (%)	**50.0**	**62.4**	**56.4**	**44.4**	**20.8**
Yes (%)	**19.8 ∗∗∗**	**30.9 ∗∗∗**	**27.7 ∗∗∗**	**23.9 ∗∗∗**	**5.6 ∗∗∗**
OR (95% CI)	**0.25 (0.17, 0.35) ∗∗∗**	**0.27 (0.19, 0.38) ∗∗∗**	**0.30 (0.21, 0.43) ∗∗∗**	**0.39 (0.28, 0.55) ∗∗∗**	**0.22 (0.12, 0.41) ∗∗∗**
aOR (95% CI)^b^	**0**.**35 (0**.**25, 0**.**50) ∗∗∗**	**0**.**43 (0**.**30, 0**.**61) ∗∗∗**	**0**.**42 (0**.**29, 0**.**60) ∗∗∗**	**0**.**50 (0**.**34, 0**.**74) ∗∗**	**0**.**45 (0**.**24, 0**.**82) ∗**
Lack of partner encouragement of PNC					
No (%)	**50**.**9**	**63**.**9**	**58**.**0**	**45**.**3**	**21**.**5**
Yes (%)	**14**.**9 ∗∗∗**	**24**.**1 ∗∗∗**	**20**.**4 ∗∗∗**	**19**.**4∗∗∗**	**2**.**1 ∗∗∗**
OR (95% CI)	**0**.**17 (0**.**11, 0**.**25) ∗∗∗**	**0**.**18 (0**.**13, 0**.**26) ∗∗∗**	**0**.**19 (0**.**12, 0**.**28) ∗∗∗**	**0**.**29 (0**.**20, 0**.**43) ∗∗∗**	**0**.**08 (0**.**04, 0**.**18) ∗∗∗**
aOR (95% CI)^b^	**0**.**24 (0**.**16, 0**.**36) ∗∗∗**	**0**.**25 (0**.**17, 0**.**37) ∗∗∗**	**0**.**26 (0**.**17, 0**.**39) ∗∗∗**	**0**.**41 (0**.**27, 0**.**62) ∗∗∗**	**0**.**16 (0**.**07, 0**.**35) ∗∗∗**
Combined lack of partner encouragement					
Encouragement of Both ANC and PNC (%)	**54**.**0**	**66**.**6**	**60**.**3**	**46**.**9**	**23**.**3**
Lack of Encouragement of ANC or PNC (%)	**25**.**7**	**39**.**5**	**35**.**3**	**29**.**9**	**5**.**6**
Lack of Encouragement of ANC and PNC (%)	**10**.**9 ∗∗∗**	**18**.**3∗∗∗**	**15**.**4 ∗∗∗**	**15**.**1 ∗∗∗**	**2**.**6 ∗∗∗**
OR (95% CI)	**0**.**29**	**0**.**33**	**0**.**36**	**0**.**48**	**0**.**19**
i. Lack of Encouragement of ANC or PNC	**(0**.**21, 0**.**41) ∗∗∗**	**(0**.**23, 0**.**47) ∗∗∗**	**(0**.**25, 0**.**52) ∗∗∗**	**(0**.**34, 0**.**69) ∗∗∗**	**(0**.**10, 0**.**36) ∗∗∗**
i. Lack of Encouragement of ANC and PNC	**0**.**10 (0**.**06, 0**.**19) ∗∗∗**	**0**.**11 (0**.**07, 0**.**18) ∗∗∗**	**0**.**12 (0**.**07, 0**.**20) ∗∗∗**	**0**.**20 (0**.**12, 0**.**33) ∗∗∗**	**0**.**09 (0**.**03, 0**.**22) ∗∗∗**
aOR (95% CI)^b^					
i. Lack of Encouragement of ANC or PNC	**0**.**37(0**.**27, 0**.**52) ∗∗∗**	**0**.**46(0**.**31, 0**.**69) ∗∗∗**	**0**.**46(0**.**32, 0**.**66) ∗∗∗**	**0.53(0.35, 0.80)∗∗**	**0**.**30(0**.**16, 0**.**57) ∗∗**
i. Lack of Encouragement of ANC and PNC	**0**.**16 (0**.**09, 0**.**29) ∗∗∗**	**0**.**19 (0**.**12, 0**.**30) ∗∗∗**	**0**.**18 (0**.**11, 0**.**30) ∗∗∗**	**0**.**30 (0**.**17, 0**.**54) ∗∗∗**	**0**.**23 (0**.**09, 0**.**59) ∗∗**

OR/aOR from svy: logistic regression model.

p-value ∗<0.05; ∗∗<0.01; ∗∗∗<0.001; bold indicates p < 0.05.

RC = reproductive coercion; IPV = intimate partner violence; ANC = antenatal care; PNC = postnatal care.

a Among women with infants still living at six-week interview (n = 1824).

b Adjusted for urban/rural residence, parity, education.

Both lack of partner encouragement of ANC and lack of partner encouragement of PNC were independently associated with decreased care-seeking at every point across the MNH continuum of care. Specifically, women whose partners did not encourage them to seek ANC reported 60% reduced odds in completing ANC (aOR = 0.35; 95% CI = 0.25–0.50; p < 0.001), delivering within a facility (aOR = 0.43; 95% CI = 0.30–0.61; p < 0.001), attending PNC by six-weeks postpartum (aOR = 0.42; 95% CI = 0.29–0.60; p < 0.001), and a 50% reduced odds of immunizing their baby at six-weeks (aOR = 0.50; 95% CI = 0.34–0.74; p = 0.01). Lack of partner encouragement of PNC was similarly associated with substantially reduced odds of complete ANC (aOR = 0.24; 95% CI = 0.16–0.36; p < 0.001), facility delivery (aOR = 0.25; 95% CI = 0.17–0.37; p < 0.001), PNC by six-weeks postpartum (aOR = 0.26; 95% CI = 0.17–0.39); and infant immunization (aOR = 0.41; 95% CI = 0.27–0.62; p < 0.001). Lack of partner encouragement of ANC (aOR = 0.45; 95% CI = 0.24–0.82; p = 0.05) and lack of partner encouragement of PNC (aOR = 0.16; 95% CI = 0.07–0.35; p < 0.001) were further associated with significant reductions in completion of the MNH continuum of care. Neither IPV nor RC were associated with completion of the continuum of care.

Lastly, we examined the dose–response relation between experience of full encouragement, lacking one type of encouragement (either ANC or PNC) and lacking encouragement for both ANC and PNC. Adjusted models revealed that lack of encouragement for both ANC and PNC contributed to the strongest reductions in care-seeking, ranging from 70% reduced odds of infant immunization (aOR = 0.30; 95% CI = 0.17–0.54; p < 0.001) to 80% reduced odds of complete ANC (aOR = 0.16; 95% CI = 0.09–0.29; p < 0.001). Compared to women who were encouraged to ANC and PNC, women whose partners did not encourage them to seek ANC or PNC had slightly greater odds of completing the MNH continuum of care (aOR = 0.30; 95% CI = 0.16–0.57; p = 0.01) than women who were not encouraged to seek either ANC or PNC (aOR = 0.23; 95% CI = 0.09–0.59; p = 0.01).

## Discussion

These findings highlight the pervasiveness of partner-related autonomy constraints prior to and during pregnancy—specifically, we found that three in five women experienced at least one of the examined autonomy constraints during this critical stage of the life course. While autonomy constraints generally occurred in isolation, all constraints were associated with reductions in care-seeking behaviors across the MNH continuum of care: RC with infant immunization; IPV during pregnancy with facility-based delivery; lack of partner encouragement of ANC or PNC with all MNH outcomes. Examination of partner dyad-level constraints and their impact is critical given that less than one in five women completed the MNH continuum of care. While previous research and interventions have largely focused on access to and quality of health services as major barriers to care-seeking, the role of the partner should not be overlooked.

Results elucidate that lack of encouragement and/or discouragement, without explicit acts of violence, may still profoundly impact the health of women and their children. Notably, most participants classified as “lacking encouragement” were not actually reporting “discouragement.” While lack of encouragement for seeking services may not be conceptualized as congruent in terms of severity with IPV and RC, these partner-related autonomy constraints were nearly twice as prevalent. Further, lack of encouragement for both ANC and PNC were associated with decreased care-seeking at every point in the MNH continuum of care, with impacts most profoundly seen for maternal health care-seeking (ANC, delivery, and PNC). Reductions in care-seeking were amplified for women who lacked support in seeking both ANC and PNC—less than 3% of women who experienced both constraints completed the continuum of care. These results speak to the profound role that partners play in women pregnancy-related care-seeking behaviors. While some research has indicated that men do not wish to be involved in women's sexual and reproductive health because they felt these are “women's issues”,^25^ in societies where men are seen as gatekeepers to health and ultimate decision-makers within the family unit, women may not feel comfortable seeking care without full support and encouragement from their partners. These concerns are further intensified in societies where women frequently lack financial autonomy and are thus reliant on partner financial support to seek care. While the present research was unable to distinguish the multiple ways in which partners could encourage or discourage care-seeking, including by verbal, financial, or physical means, we encourage future research and programs to measure and evaluate the role of partner support in care-seeking behaviors on a spectrum, rather than only assessing full discouragement or encouragement.

For women within abusive relationships, pregnancy and the postpartum period are critical times for intervention due to increased contact with healthcare providers. Our findings underscore that for women experiencing IPV during pregnancy, however, a focus on the postnatal period may miss a substantial number of women, given the significantly lower rates of facility-based delivery among women experiencing IPV (57.5% facility delivery among those without IPV during pregnancy versus 39.6% facility delivery among those with IPV during pregnancy). Previous research has found that experiencing IPV decreased the receipt of timely ANC,^26^ however, our results indicate that IPV during pregnancy did not significantly impact receipt of complete ANC. These results speak to ANC as a critical time for IPV screening and integration of safety planning to ensure women and health systems recognize danger signs prior to severe violence and know pathways to help-seeking, if danger should present. Given reductions in facility-based delivery for women experiencing IPV during pregnancy, ANC visits may be one of the only points of contact with formal care services—training on trauma-informed screening, safety planning, and response is imperative for these providers. Trainings such as those from the World Health Organization, which emphasize L.I.V.E.S—Listen, Inquire, Validate, Enhance safety, and Support—are critical in teaching providers to respond to 10.13039/501100014193IPV in a non-stigmatizing, supportive manner.^27^ These trainings were piloted in Ethiopia in February 2022 and should be expanded upon further evaluation.^28^ ANC visits should further serve as an opportunity to discuss barriers to facility delivery and ways to engage partners in birth plans and care-seeking.

We were surprised to find that RC was not significantly related to any of the maternal health-seeking behaviors, but instead was associated with a nearly 50% reduction in odds of infant immunization. These results may point to pronatalist male partners seeking to ensure a healthy pregnancy and delivery; however, such hypotheses were not directly explored in this analysis and do not support the observed reductions in infant immunization. RC has been linked to child developmental outcomes within the United States, however, care-seeking behaviors (e.g., infant immunization) were not examined^29^; there is a void of research understanding the impact of RC on child development and subsequent pregnancies globally and specifically within sub-Saharan Africa. Future work should focus on how abusive and coercive behaviors prior to pregnancy continue to hinder women's care-seeking for themselves and their children, as well as how these associations may be impacted by social norms, including potential vaccine hesitancy, at the partnership level.

These results are not without limitations. Partner-related autonomy constraints occurred in relative isolation—as such, the cumulative effect of experiencing multiple constraints could only be assessed for lack of encouragement of ANC and PNC. Correlations between partner-related autonomy constraints were presented to disentangle some of these associations, however, given that all constraints were measured at six-week (or baseline for RC), future research is needed to understand temporality and potential cumulative impact of these constraints. Further, prevalence of actual partner discouragement of ANC and PNC were small (<1%) and thus combined with “lack of encouragement” for further assessment—accordingly, these measures better assess lack of encouragement rather than overt discouragement. Qualitative data, including from the male partner, are needed to better understand the perceived roles and motivations of partners in encouraging or discouraging care-seeking to ultimately understand potential pathways to improve engagement. Moreover, quantitative data specific to the male partner are limited. Our analysis did not account for the potential role of health systems in women's completion of recommended care—for example, access to and receipt of high-quality care from pre-pregnancy throughout the postpartum period—which may shape women's continued pursuit of health services during this critical time.

In Ethiopia, less than half of women deliver within facilities, complete ANC, obtain one PNC check, or have their newborn fully immunized per global recommendations—less than one in five women complete all of these vital steps. This longitudinal research elucidates the interplay of partner-related autonomy constraints, and their associations with MNH outcomes to inform interventions at the couple and provider levels and women-centered messaging tailored to individual circumstances. Partner encouragement is needed for women to succeed in seeking the care they need during and after pregnancy—notably, combatting discouragement is not enough. Programs must not only invest in counteracting partners’ harmful beliefs surrounding help-seeking, but also invest resources in giving men the knowledge and tools they need to support their partners throughout their pregnancy. Interventions such as Charm2 have been successful in engaging couples, as individuals and together, to enhance gender equity and family planning^30^—such programs could be extended to engage couples in wider reproductive health behaviors, including ANC, pregnancy, and postnatal care. Women experience disproportionate burdens on their health and that of their children when they are unable to receive timely, critical MNH care. Policies and programs that seek to increase gender equity and reduce morbidity and mortality must engage men to be supporting partners, while also ensuring women have access to necessary information and actionable resources to circumvent potential barriers to care imposed by partners.

## Contributors

Shannon N. Wood: literature search, accessed raw data, data analysis, data interpretation, writing.

Robel Yirgu: data interpretation, literature search, writing.

Celia Karp: data interpretation, accessed raw data, data analysis, writing.

Meseret Zelalem Tadesse: data interpretation, writing.

Solomon Shiferaw: study design, data collection, data interpretation, writing.

Linnea A. Zimmerman: study design, data interpretation, writing.

All authors read and approved the final version of this manuscript.

## Data sharing statement

Data are available upon request from www.pmadata.org.

## Declaration of interests

Shannon Wood, Celia Karp, and Linnea Zimmerman report support for the present manuscript and attending meetings and/or travel from the 10.13039/100000865Bill and Melinda Gates Foundation, with payment to 10.13039/100007880Johns Hopkins University.
